# Fcγ Receptor-Dependent Internalization and Off-Target Cytotoxicity of Antibody-Drug Conjugate Aggregates

**DOI:** 10.1007/s11095-021-03158-x

**Published:** 2021-12-27

**Authors:** Michihiko Aoyama, Minoru Tada, Hidetomo Yokoo, Yosuke Demizu, Akiko Ishii-Watabe

**Affiliations:** 1 Division of Biological Chemistry and Biologicals, National Institute of Health Sciences, 3-25-26 Tonomachi, Kawasaki-ku, Kawasaki, Kanagawa 210-9501 Japan; 2 Division of Organic Chemistry, National Institute of Health Sciences, 3-25-26 Tonomachi, Kawasaki-ku, Kawasaki, Kanagawa 210-9501 Japan

**Keywords:** Antibody-drug conjugates, aggregation, off-target toxicity, Fcγ receptors

## Abstract

**Purpose:**

Antibody-drug conjugates (ADCs), which are monoclonal antibodies (mAbs) conjugated with highly toxic payloads, achieve high tumor killing efficacy due to the specific delivery of payloads in accordance with mAbs’ function. On the other hand, the conjugation of payloads often increases the hydrophobicity of mAbs, resulting in reduced stability and increased aggregation. It is considered that mAb aggregates have potential risk for activating Fcγ receptors (FcγRs) on immune cells, and are internalized into cells via FcγRs. Based on the mechanism of action of ADCs, the internalization of ADCs into target-negative cells may cause the off-target toxicity. However, the impacts of aggregation on the safety of ADCs including off-target cytotoxicity have been unclear. In this study, we investigated the cytotoxicity of ADC aggregates in target-negative cells.

**Methods:**

The ADC aggregates were generated by stirring stress or thermal stress. The off-target cytotoxicity of ADC aggregates was evaluated in several target-negative cell lines, and FcγR-activation properties of ADC aggregates were characterized using a reporter cell assay.

**Results:**

Aggregation of ADCs enhanced the off-target cytotoxicity in several target-negative cell lines compared with non-stressed ADCs. Notably, ADC aggregates with FcγR-activation properties showed dramatically enhanced cytotoxicity in FcγR-expressing cells. The FcγR-mediated off-target cytotoxicity of ADC aggregates was reduced by using a FcγR-blocking antibody or Fc-engineering for silencing Fc-mediated effector functions.

**Conclusions:**

These results indicated that FcγRs play an important role for internalization of ADC aggregates into non-target cells, and the aggregation of ADCs increases the potential risk for off-target toxicity.

**Supplementary Information:**

The online version contains supplementary material available at 10.1007/s11095-021-03158-x.

## Introduction

Antibody-drug conjugates (ADCs), which are monoclonal antibodies (mAbs) conjugated with highly toxic small molecules (payloads) via linkers, are one of the fastest growing classes of next generation mAbs. ADCs combine the advantages of the target-specificity of mAbs with the high tumor killing efficacy of payloads. Namely, ADCs are specifically transported to the cells expressing their target antigens in accordance with the function of mAbs, and the ADCs are internalized and subsequently release the payloads to kill the target cells. Therefore, it is expected that ADCs will reduce the systemic exposure of cytotoxic small molecules while providing a wider therapeutic window compared with traditional chemotherapy. The development and commercial application of ADCs have been progressing in recent years. Six of ten FDA-approved ADCs were approved since the start of 2019, and 85 candidates are at the clinical development stage in various countries (1).

Though ADCs have great advantages for cancer therapy, there are some ADC-specific problems resulting from particular characteristics of ADCs. One of the problems is the increase in hydrophobicity due to the conjugation of the hydrophobic payload to mAbs. Though mAbs naturally have a hydrophilic character, most of the payloads are too hydrophobic, and conjugation of payloads to mAbs often increases the hydrophobicity. The hydrophobicity of ADCs is affected by the drug antibody ratio (DAR) and characteristics of the linker and payload, and it is well known that the hydrophobicity of ADCs affects the plasma clearance and therapeutic index (2–4). In addition, the increase of surface hydrophobicity induced by conjugation of hydrophobic payloads promotes the aggregation of ADCs followed by enhancement of non-specific protein interactions in the drug products (5). Thus, the aggregation rate of ADCs was often higher than that of the native mAbs (5, 6). In biopharmaceuticals, including ADCs, protein aggregates are believed to be key risk factors for immunogenicity (7). Therefore, aggregation of ADCs via an increase of hydrophobicity has been well studied in the development of ADC formulations.

In therapeutic mAbs, some reports have indicated that the mAb aggregates could enhance immunogenicity through the activation of immune cells via Fcγ receptors (FcγRs) (8–10). In addition, it was reported that mAb aggregates showed higher internalization properties compared with native mAbs, and quickly accumulated at the degradation pathways involving late endosomes in mouse dendritic cells (11). Thus, mAb aggregates could not only activate immune cells via the receptors on the cell surface but could also be internalized into the cells which did not express the target antigen. Considering the mechanism of action of ADCs, unintended cellular uptake and accumulation at the degradation pathway of ADCs in non-target cells may cause the off-target toxicity (12, 13). However, the impact of aggregation on the safety of ADCs, especially off-target toxicities induced by unintended internalization into non-target cells, has been unclear.

In this study, we evaluated the impact of ADC aggregation on cytotoxicity in target-positive and -negative cells using two commercially available ADCs, and revealed that the aggregation of ADCs reduced the target-dependent cytotoxicity but also increased the target-independent cytotoxicity in several cell lines. We also indicated that the enhancements of target-independent cytotoxicity of ADC aggregates were related to FcγR-mediated cellular uptake of ADC aggregates. Our results revealed that the aggregation of ADCs increases the potential risk of not only immunogenicity but also adverse effects via off-target cytotoxicity of ADCs.

## Materials and Methods

### Cell Line

TMNK-1 cells (an immortalized human liver endothelial cell line, JCRB1564), MEG-01 s cells (a human megakaryoblastic leukemia cell line, IFO50473), and THP-1 cells (a human acute monocytic leukemia cell line, JCRB0112) were obtained from the JCRB cell bank (Osaka, Japan). Jurkat cells (a human T lymphocyte cell line, RCB0806) were obtained from the RIKEN BRC. SK-BR-3 cells (an HER2^+^ human breast cancer cell line, ATCC® HTB-30) was obtained from ATCC. Jurkat cells expressing human FcγRIIa or FcγRIIIa with the Nuclear Factor of Activated T cells (NFAT)-driven luciferase reporter (Jurkat/FcγRIIa/NFAT-Luc, Jurkat/ FcγRIIIa/NFAT-Luc) were established previously (14, 15). TMNK-1 cells were maintained in Medium 200 (Thermo Fisher Scientific, Waltham, MA) supplemented with Low Serum Growth Supplement (Thermo Fisher Scientific) at 37°C in a humidified atmosphere containing 5% CO_2_. Other cell lines were maintained in RPMI1640 medium supplemented with 10% FBS at 37°C in a humidified atmosphere containing 5% CO_2_. The expression of the FcγRs in the cell lines was measured by FITC-labelled anti-CD64 antibody, anti-CD32 antibody, and anti-CD16 antibody (BD Biosciences; San Jose, CA) using BD FACSCanto II (BD Biosciences).

### Monoclonal Antibody and Antibody-Drug Conjugates

Trastuzumab (Herceptin®) and trastuzumab emtansine (T-DM1, Kadcyla®) were purchased from Chugai Pharmaceutical (Tokyo). Trastuzumab deruxtecan (T-DXd, Enhertu®) was purchased from Daiichi Sankyo (Tokyo). DNA fragments encoding the trastuzumab heavy-chain and light-chain were synthesized by TaKaRa Bio (Kusastu, Japan), and subcloned into pFUSE-CHIg-hG1 and pFUSE2-CLIg-hk vector (InvivoGen; San Diego, CA), respectively. The expression vectors encoding anti-HER2 mAbs with L234A/L235A substitutions were constructed using synthesized DNA fragments as described previously (14). Recombinant anti-HER2 mAbs were produced using the Expi293™ Expression System (Thermo Fisher Scientific) according to the manufacturer’s instructions, and the products were purified using a HiTrap Protein G HP column (Cytiva; Marlborough, MA).

Anti-HER2 mAb-based ADCs and Alexa488-labelled trastuzumab (Tra-FL) were generated as described below. The anti-HER2 mAbs or trastuzumab were reduced with 2.2 or 6 equivalents of TCEP in PBS with 50 mM sodium borate and 1 mM DTPA for 2 h incubation at 37°C, and labelled by adding 5 equivalents of mc-MMAE (Chemscene; Monmouth Junction, NJ) or 10 equivalents of Alexa Fluor™ 488 C5 Maleimide (Thermo Fisher Scientific) at 4°C, respectively. The excess mc-MMAE or dye reagent was removed by using a PD-10 desalting column (Cytiva). The protein concentration of the anti-HER2 mAb-based ADCs and Tra-FL and the number of dyes per antibody of Tra-FL were calculated from the absorbance measured using a Nanodrop 2000c spectrophotometer (Thermo Fisher Scientific). The DAR values of the anti-HER2 mAb-based ADCs were analyzed by hydrophobic interaction chromatography (HIC). HIC analysis was performed by using an Agilent 1100LC system (Agilent, Santa Clara, CA) with a TSK-gel Butyl-NPR column (4.6 mm × 10 cm, 2.5 μm particle size; Tosoh Bioscience, Tokyo) with a linear gradient of 100% mobile phase A [1.5 M (NH_4_)_2_SO_4_ in 25 mM potassium phosphate] to 100% mobile phase B (25 mM potassium phosphate, pH 7.0 + 25% isopropanol) in 40 min, and chromatograms were obtained by a UV detector (280 nm). Anti-CD32 antibody (IV.3) was purified from the supernatant of a hybridoma obtained from ATCC (HB-217) using a HiTrap Protein G column (Cytiva), and the Fab fragment was prepared by using a Fab Preparation Kit (Thermo Fisher Scientific).

### Aggregate Preparation and Size Distribution Analysis

Aggregates were generated by stirring or thermal stress. For the stirring stress, 2 mL of mAb or ADC solution diluted in PBS (Thermo Fisher Scientific) (0.375 mg/mL, 2.5 μM) was stirred with a 1.5 × 8.0 mm PTFE stirrer bar in a 6 mL glass vial at ~600 rpm for 20 h. For the thermal stress, 1 mL of mAb or ADC solution diluted in PBS (0.375 mg/mL, 2.5 μM) was incubated at 90°C for 3 min followed by incubation on ice for over 15 min. Filtration of the aggregate solution was performed with a MILLEX®-HV 0.45 μm filter unit (Merck Millipore, Burlington, MA). The size distribution of the mAb or ADC aggregates was analyzed using a quantitative laser diffraction (qLD) method as described previously (10). The samples were 10-fold diluted in prefiltered PBS, and analyzed using an Aggregates Sizer SALD-7500 nano with WingSALD bio version 3.3 software (Shimadzu Corporation, Kyoto, Japan). The density of 1.37 g/cm^3^ was used to convert the volume distribution into the weight distribution.

### Size Exclusion Chromatography (SEC)

SEC analysis was performed by using AKTA Avant 25 and Superdex 200 Increase 10/300GL column (Cytiva). The samples (500 μL) were injected into the column at a flow rate of 0.75 ml/min in PBS. The relative amount (%) of high molecular weight (HMW) species, monomer, and fragments against total peak area of control samples were calculated from peak area of chromatogram.

### Flow Imaging (FI)

FI analysis was performed by using FlowCam 8100 (Fluid Imaging Technologies, Inc., Scarborough, ME) as previously described (16). The samples were 200-fold diluted in prefiltered PBS, and the flow rate, sample volume, autoimage rate, and segmentation threshold (dark/light) were set for 0.1 mL/min, 0.2 mL, 17 frames/s, and 10/10, respectively. The results of three samples from different preparation were collected and analyzed. The counts of particles were obtained from the particle numbers in the ranges of 2.0 ~ 100 μm (area-based diameter, ABD).

### Proliferation Assay

TMNK-1 cells and SK-BR-3 cells were seeded at 2 × 10^3^ cells/well in 96-well tissue-culture plates (Iwaki; Tokyo), and incubated overnight at 37°C in a humidified atmosphere containing 5% CO_2_. Jurkat cells, MEG01-S cells, THP-1 cells, and Jurkat/FcγRs/NFAT-Luc cells were seeded at 1 × 10^4^ cells/well in non-treated 96-well plates (Iwaki). FcγRIIa blocking was performed by pre-incubation for 30 min with 2 μg/mL of Fab fragment of anti-FcγRIIa antibody clone IV.3 (IV.3-Fab). Then serially diluted samples were added to each well, followed by incubation at 37°C in a humidified atmosphere containing 5% CO_2_ for 3 days. After 3 days of incubation, the cell proliferation was evaluated by WST-8 assay using Cell Count Reagent SF (Nakarai Tesque, Kyoto, Japan). The data were analyzed by using GraphPad Prism7 software (GraphPad Software, San Diego, CA). The IC_50_ values of ADC and ADC aggregates were calculated with 4 parameter logistic curve fitting.

### FcγR Reporter Assay

The Jurkat/FcγRs/NFAT-Luc cells were washed and suspended in Opti-MEM™ I Reduced Serum Medium (Thermo Fisher Scientific), and were seeded into a 96-well round-bottom plate (1 × 10^5^ cells/well). Then serially diluted samples were added to each well, followed by incubation at 37°C in a humidified atmosphere containing 5% CO_2_ for 4 h. Luciferase activity was measured by using a ONE-Glo Luciferase Assay Reagent (Promega, Madison, WI) and Ensight multimode plate reader (PerkinElmer, Waltham, MA).

### FcγR-Mediated Internalization of Trastuzumab-Alexa488 Aggregate

The Jurkat/FcγRs/NFAT-Luc cells were washed and suspended in Opti-MEM™ I Reduced Serum Medium (Thermo Fisher Scientific), and were seeded into a 96-well round-bottom plate (1 × 10^5^ cells/well). The cells were then incubated at 37°C or 4°C for 30 min. The FcγRIIa blocking was performed by pre-incubation for 30 min with 2 μg/mL of IV.3-Fab at 37°C. The 2.5-fold serially diluted Alexa488-labelled trastuzumab samples (4 points; 2-80 nM final concentration) were added to each well, followed by incubation at 37°C or 4°C for 4 h. The cells were washed twice with PBS, and resuspended with stain buffer (PBS with 0.5% BSA, 2 mM EDTA, 0.1% NaN_3_). The fluorescence intensities of the cells were analyzed by a FACSCanto II flow cytometer.

## Results

### The Cytotoxicity of ADC Aggregates

**Fig. 1:**
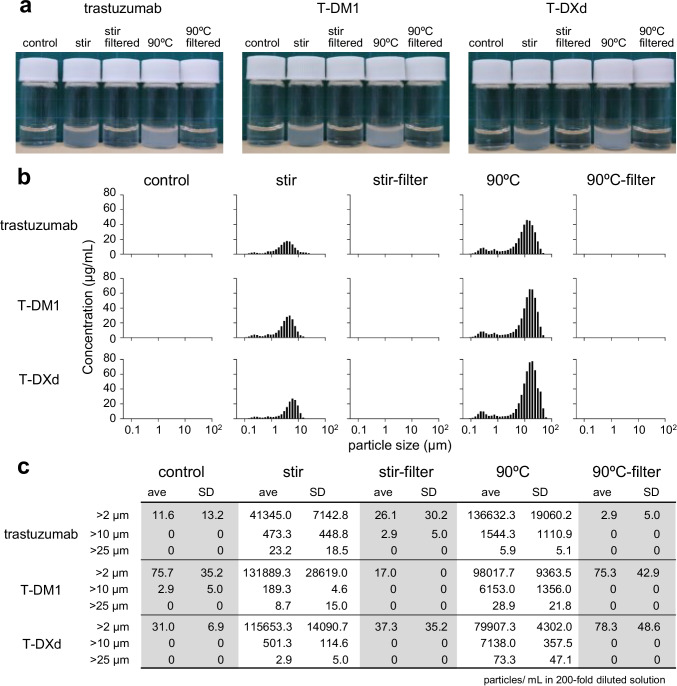
The characterization of ADC aggregates induced by stirring stress or thermal stress. Aggregates of trastuzumab and commercial ADCs (T-DM1, T-DXd) were prepared by stirring stress (~600 rpm, 20 h) or thermal stress (90°C, 3 min) in PBS, and then an aliquot of the sample was filtered by using a 0.45 μm filter. (**a**) Pictures of 0.375 mg/mL control (non-stressed) and stressed samples. (**b**) The size distribution of 10-fold diluted sample was analyzed by using qLD. Representative data are presented. (**c**) Particle counts in 200-fold diluted samples obtained by Flow Imaging. Value is the mean and standard deviation of three samples from different preparations.

First, we examined whether aggregation of ADCs affects the cytotoxicity of ADCs in target cells and non-target cells. In this study, we chose three commercial pharmaceuticals, an anti-HER2 mAb (trastuzumab), and two trastuzumab-based ADCs with different linker-payloads, T-DM1 and T-DXd. T-DM1 is composed of a maytansine derivative DM1(microtubule inhibitor) conjugated to lysine residues of trastuzumab via a stable thioether (17). T-DXd is composed of an exatecan derivative DXd (DNA topoisomerase I inhibitor) conjugated to reduced cysteine residues via a cleavable peptide (GGFG) linker (18). These mAb and ADCs have killing activities against HER2-positive cancer cells and are approved for HER2-positive cancer therapy. We generated the aggregates of trastuzumab, T-DM1, and T-DXd by stirring stress (600 rpm, 20 h) or thermal stress (90°C, 3 min) in this study. As shown in Fig. 1a, stirring- and thermal stress induced high level aggregation of trastuzumab, T-DM1, and T-DXd, and the solutions became clouded by aggregates which could be removed by 0.45 μm filter. We performed size distribution analysis and particle counts for characterizing these aggregates. As shown in Fig. 1b, a qLD size distribution analysis showed that aggregates between 0.1 and 50 μm were generated by stirring stress and thermal stress in each of trastuzumab, T-DM1, and T-DXd. Though the profiles of the size distribution of ADC aggregates differed between the induction methods (thermal stress and stirring stress), the aggregates induced by the same type of stress showed a similar profile regardless of whether they were mAbs or ADCs. In addition, after filtration of ADC aggregates with a 0.45 μm filter, the aggregated proteins in filtered samples were reduced to a level similar to those in the control (non-stressed) groups. As the result of FI analysis, the stirring- or thermal-stress treated samples contained a lot of particles with over 2 μm area-based diameter while control (non-stressed) or 0.45 μm filtered samples contained few particulate matters (Fig. 1c). We next performed SEC analysis to characterize the remained ADCs in the filtrated samples (Supplementary Fig. S1). As the results, >75% of ADCs in stressed samples were removed by 0.45 μm filter, and most of remained ADCs were monomeric though filtered samples of T-DXd aggregates contained some fragments.

**Fig. 2:**
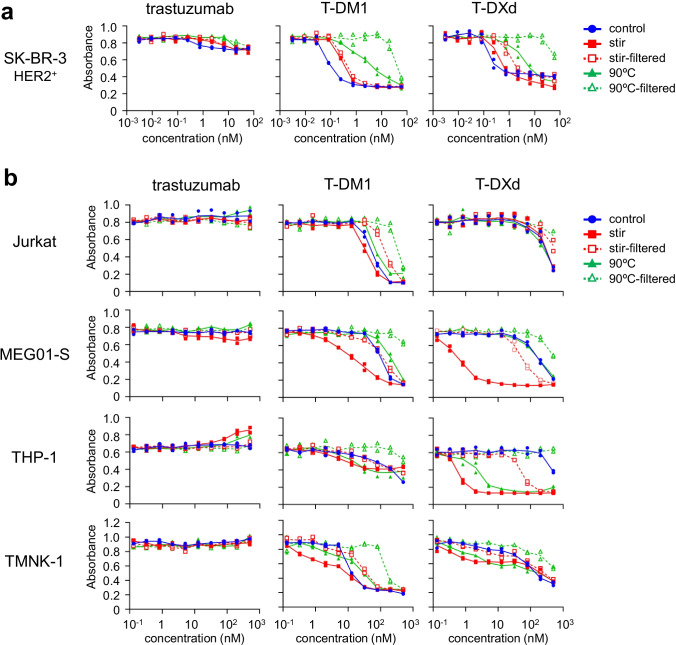
The cytotoxicity of ADC aggregates. (**a**) The cytotoxicity of aggregated ADCs in HER2-positive cells. (**b**) The cytotoxicity of aggregated ADCs in HER2-negative cells. The HER2-positive cells (SK-BR-3) or HER2-negative cells (Jurkat, MEG-01S, THP-1, TMNK-1) were incubated with serially diluted samples (control, aggregates, or filtrated samples) for 3 days, and the cell proliferations were measured by WST-8 assay. The concentrations of filtered samples represent pre-filtration concentration of the samples. The data represent individual plots (n = 2).

To evaluate the impacts of aggregation, we assessed the cytotoxicity of control (non-stressed) ADCs and ADC aggregates in HER2 (target antigen)-positive cells. As shown in Fig. 2a, the control trastuzumab, T-DM1, and T-DXd showed dose-dependent cytotoxicity in an HER2-positive cell line (SK-BR-3), and the aggregates of trastuzumab, T-DM1, and T-DXd showed lower cytotoxicity compared with the control group regardless of whether they were induced by stirring stress or thermal stress. The cytotoxicity of filtered samples of ADC aggregates was equal to or lower than that of ADC aggregates before filtration. These data indicated that the aggregation of ADCs reduced cytotoxicity to target cells.

Next, we assessed the cytotoxicity of ADC aggregates in several HER2-negative cell lines—Jurkat cells (a human T lymphocyte cell line), MEG-01 s cells (a human megakaryoblastic leukemia cell line), THP-1 cells (a human acute monocytic leukemia cell line), and TMNK-1 cells (an immortalized human liver endothelial cell line)—. As shown in Fig. 2b, trastuzumab and the aggregates induced by stirring- and thermal stress did not show any cytotoxicity in HER2-negative cell lines. The control T-DM1 and T-DXd showed >100-fold less cytotoxicity than SK-BR-3 cells regardless of the cell line (Supplementary Table S1). On the other hand, the aggregation of ADCs had a different impact on the cytotoxicity in HER2-negative cell lines. In Jurkat cells, the stirring stress-induced T-DM1 aggregate showed slightly higher cytotoxicity than T-DM1, while both the stirring stress- and thermal stress-induced T-DXd aggregates showed cytotoxicity similar to that of control T-DXd. In MEG01-S cells, stirring stress-induced aggregates of T-DM1 and T-DXd showed higher cytotoxicity than the control group, while thermal stress-induced aggregates showed the same or lower cytotoxicity compared with the control group. In THP-1 cells, ADC aggregates, especially T-DXd aggregates induced by stirring stress or thermal stress, showed higher cytotoxicity than control ADCs. In TMNK-1 cells, both T-DM1 and T-DXd aggregates showed enhanced cytotoxicity at low ADC concentration regardless of the aggregation method (stirring stress or thermal stress). These data indicated that aggregation of ADCs could enhance the cytotoxicity in non-target cells, particularly in the case of the stirring stress-induced T-DXd aggregates, which showed a > 100-fold increase in cytotoxicity compared with control T-DXd in MEG01-S cells and THP-1 cells (IC_50_ in MEG01-S: control 194.8 nM, stir 0.6 nM; IC_50_ in THP-1: control 498.6 nM, stir 0.6 nM). Furthermore, the filtration of ADC aggregates reduced the increase in cytotoxicity induced by aggregation regardless of cell lines, ADCs, or type of stress, suggesting that at least a part of the enhancement of cytotoxicity by aggregation was induced by aggregates that were removed by the 0.45 μm filter. Our results revealed that the aggregation of ADCs not only reduces the killing activity in target cells but also enhances the off-target toxicity in non-target cells.

### FcγR-Activation Properties and Cytotoxicity of ADC Aggregates

**Fig. 3:**
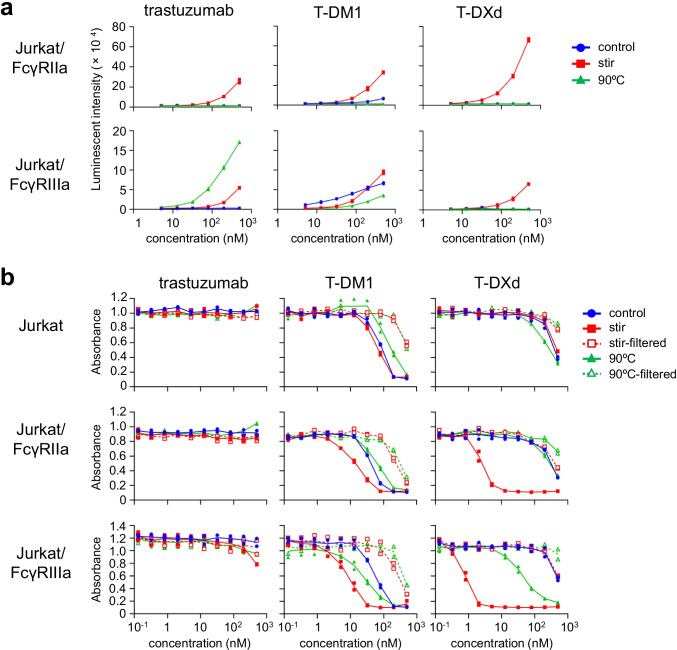
FcγR-activation and cytotoxicity of ADC aggregates in FcγR-expressing reporter cells. (**a**) The FcγR-activation properties of ADC aggregates in FcγR-expressing reporter cells. FcγR-expressing reporter cells were incubated with serially diluted samples (control or aggregates) for 4 h, and the luciferase activities were measured. (**b**) The cytotoxicity of ADC aggregates in FcγR-expressing reporter cells. FcγR-expressing reporter cells were incubated with serially diluted samples (control, aggregates, or filtrated samples) for 3 days, and the cell proliferations were measured by WST-8 assay. The concentrations of filtered samples represent pre-filtration concentration of the samples. The data represent individual plots (n = 2).

The cytotoxicity of ADC aggregates was different among the HER2-negative cell lines and dramatically enhanced in MEG01-S cells and THP-1 cells. These cell lines derived from immune cells (megakaryocytes or monocytes) expressed the FcγRs (supplementary Fig. S2). Therefore, we focused on the relationship between FcγRs and cytotoxicity of ADC aggregates to unravel the mechanism by which the cytotoxicity of ADCs induced by aggregation was enhanced. To evaluate the FcγR-activation properties of ADC aggregates, we performed the reporter assays using Jurkat/FcγRs/NFAT-Luc reporter cell lines which we previously developed (14, 15). As shown in Fig. 3a, the stirring stress- or thermal stress-induced aggregates of trastuzumab, T-DM1, and T-DXd showed different FcγR-activation properties. Trastuzumab-, T-DM1-, and T-DXd-aggregates induced by stirring stress activated FcγRIIa- and FcγRIIIa-expressing reporter cells, and trastuzumab- and T-DM1-aggregates induced by thermal stress activated FcγRIIIa but not FcγRIIa, whereas thermal stress-induced T-DXd aggregates did not activate FcγR-expressing reporter cells. Next, we assessed the cytotoxicity of ADC aggregates in FcγR-expressing reporter cells. As shown in Fig. 3b, control trastuzumab and aggregated trastuzumab did not show significant cytotoxicity in FcγR-expressing reporter cells. On the other hand, the stirring stress-induced ADC aggregates showed quite higher cytotoxicity than control ADCs in FcγRIIa- and FcγRIIIa-expressing reporter cells, while these aggregates did not show enhancement of cytotoxicity in Jurkat cells. The thermal stress-induced ADC aggregates showed the enhancement of cytotoxicity in FcγRIIIa-expressing reporter cells, while those aggregates did not affect the cytotoxicity in FcγRIIa-expressing cells. Furthermore, these enhancements of cytotoxicity were reduced by filtration of ADC aggregates with a 0.45 μm filter. These data indicated that the ADC aggregates with FcγR-activation properties contributed to the enhanced cytotoxicity in FcγR-expressing cells.

### FcγR-Dependent Cellular Uptake of mAb Aggregates

**Fig. 4:**
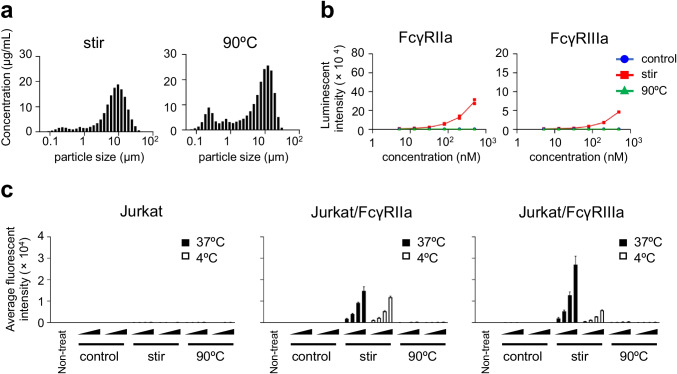
The cellular uptake of fluorescent labelled-trastuzumab aggregates in FcγR-expressing reporter cells. (**a**) The size distribution of the maleimide-C5-Alexa488-labelled trastuzumab (Tra-FL) aggregates. Tra-FL aggregates were prepared by stirring stress (~600 rpm, 20 h) or thermal stress (90°C, 3 min) in PBS. The size distribution of the sample was analyzed by using qLD. Representative data are presented. (**b**) The FcγR-activation properties of Tra-FL aggregates in FcγR-expressing reporter cells. FcγR-expressing reporter cells were incubated with serially diluted samples (control or aggregates) for 4 h, and the luciferase activities were measured. The data represent individual plots (n = 2). (**c**) The cellular uptake of Tra-FL aggregates in FcγR-expressing reporter cells. The FcγR-expressing reporter cells were incubated with serially diluted samples (control or aggregates; final antibody concentration, 5, 13, 32, or 80 nM) for 4 h at 37°C or 4°C. The fluorescent intensities were measured by a flow cytometer. The data represent the mean ± standard deviation (n = 3).

To determine whether the FcγRs were related to the cellular uptake of mAb aggregates, we evaluated the cellular uptake of Tra-FL aggregates in FcγR-expressing cells. We produced a Tra-FL using the maleimide-thiol conjugation method, and confirmed that the Tra-FL had 7.0 dyes per antibody. As shown in Fig. 4a, the stirring stress and thermal stress generated Tra-FL aggregates of between 0.1 and 50 μm with different size distribution profiles. The stirring stress-induced Tra-FL aggregates showed FcγRIIa- and FcγRIIIa-activation properties, while the thermal stress-induced aggregates did not activate the FcγRIIa- and FcγRIIIa-expressing reporter cells (Fig. 4b). These Tra-FL aggregates with different FcγR-activation properties were added to FcγR-expressing reporter cells, and the internalization of Tra-FL aggregates after 4 h of incubation was evaluated by flow cytometry analysis. As shown in Fig. 4c, the fluorescent intensities of stirring stress-induced Tra-FL aggregates-treated FcγRIIa-or FcγRIIIa-expressing cells were much higher than those of control Tra-FL or thermal stress-induced Tra-FL aggregates-treated cells. In addition, incubation under a low temperature (4°C) condition which inhibited the endocytosis pathway reduced the fluorescent intensities of stirring stress-induced Tra-FL aggregates-treated cells. These results suggested that stirring stress-induced Tra-FL aggregates, which could activate FcγRIIa- or FcγRIIIa-expressing reporter cells, were internalized into non-target cells by FcγR-dependent cellular uptake.

### Contribution of FcγRIIa to the Cytotoxicity of ADC Aggregates in MEG01-S Cells

**Fig. 5:**
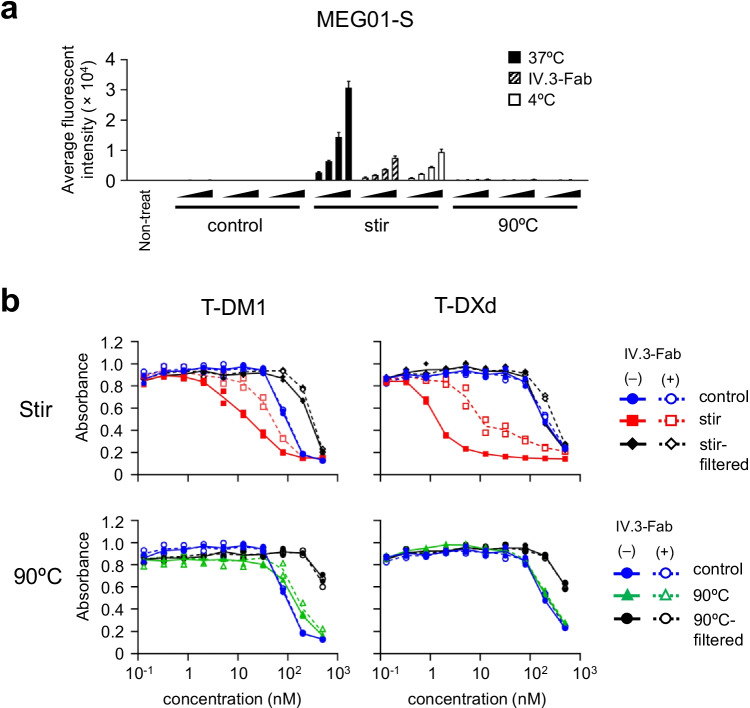
Influence of FcγRIIa-blocking on the cytotoxicity of ADC aggregates in MEG01-S cells. FcγRIIa on MEG01-S cells were blocked by using a Fab fragment of anti-FcγRIIa antibody (Fab-IV.3). MEG01-S cells were pre-treated with Fab-IV.3 (final concentration: 2 μg/mL) for 30 min, and then the serially diluted samples were added to the FcγRIIa-blocking MEG01-S cells. (**a**) The cellular uptake of Tra-FL aggregates under an FcγRIIa-blocking condition. The control cells, FcγRIIa-blocking cells, and cells under a low temperature condition were incubated with the serially diluted samples (control or aggregates; final concentration: 5, 13, 32, or 80 nM) for 4 h. The fluorescent intensities were measured by flow cytometer. The data represent the means ± standard deviation (n = 3). (**b**) The cytotoxicity of ADC aggregates under an FcγRIIa-blocking condition. The control cells and FcγRIIa-blocking cells were incubated with serially diluted samples (control, aggregates, or filtrated samples) for 3 days, and the cell proliferations were measured by wst-8 assay. The concentrations of filtered samples represent pre-filtration concentration of the samples. The data represent individual plots (n = 2).

Next, we assessed the contribution of FcγRs to the cellular uptake and cytotoxicity of ADC aggregates in naturally FcγR-expressing cells to determine whether or not this contribution was limited to forced-expression reporter cells. For this purpose, we chose the FcγRIIa-positive functional human megakaryoblastic leukemia cell line, MEG01-S. We blocked FcγRIIa on MEG01-S cells by using IV.3-Fab, and evaluated the cellular uptake and cytotoxicity of ADC aggregates. As shown in Fig. 5a, the MEG01-S cells treated with stirring stress-induced Tra-FL aggregates which activated FcγRs showed dramatically higher fluorescent intensity than the cells treated with control (non-stressed) Tra-FL or thermal stress-induced Tra-FL aggregates which did not activate FcγRs. In addition, we revealed that the FcγRIIa-blocking by IV.3-Fab decreased the fluorescent intensity of stirring stress-induced Tra-FL aggregates-treated cells to the same level as low temperature incubation. This result suggested that FcγRIIa on MEG01-S cells contributed to the cellular uptake of Tra-FL aggregates. Next, we evaluated the effects of FcγRIIa-blocking on the cytotoxicity of ADC aggregates in MEG01-S cells. As shown in Fig. 5b, the enhancement of cytotoxicity in stirring stress-induced ADC aggregates was partially suppressed by FcγRIIa-blocking, while the cytotoxicity of control (non-stressed) ADCs, thermal stress-induced aggregates, and filtrated samples was not affected by FcγRIIa-blocking. These results indicated that FcγRIIa on MEG01-S cells at least partially contributed to the uptake and cytotoxicity of ADC aggregates which could activate FcγRIIa, while FcγRIIa did not contribute to the uptake of monomeric ADCs.

### The Cytotoxicity of Fc-Engineered ADC Aggregates

**Fig. 6:**
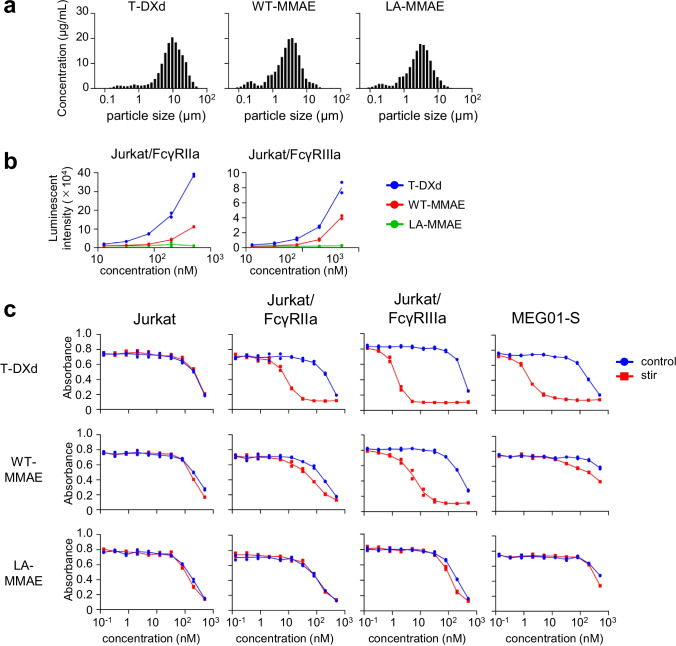
FcγR-activation and cytotoxicity of ADC aggregates with Fc-engineering in FcγR-expressing reporter cells. (**a**) The size distribution of T-DXd aggregates and anti-HER2 mAb-based ADC aggregates with different Fc regions (wildtype or L234A/L235A mutation). The aggregates were prepared by stirring stress (~600 rpm, 20 h) in PBS. The size distribution of the sample was analyzed by using qLD. Representative data are presented. (**b**) The FcγR-activation properties of T-DXd and anti-HER2 mAb-based ADC aggregates in FcγR-expressing reporter cells. FcγR-expressing reporter cells were incubated with serially diluted samples for 4 h, and the luciferase activities were measured. The data represent individual plots (n = 2). (**c**) The cytotoxicity of anti-HER2 mAb-based ADC aggregates in FcγR-expressing reporter cells and MEG01-S cells. The cells were incubated with serially diluted samples (control or stirring stress-induced aggregates) for 3 days, and the cell proliferations were measured by WST-8 assay. The data represent individual plots (n = 2).

In a previous study, we revealed that FcγR-activation of mAb aggregates depended on the Fc function of native mAbs (e.g., IgG subclass and amino acid substitutions) (10). For example, the aggregates of the L234A/L235A mutant, which was silent for FcγR-mediated effector functions (19), showed quite weaker FcγRIIa- and FcγRIIIa-activations than those of wild-type IgG1. Thus, we considered that Fc-silencing could modulate the FcγR-dependent off-target cytotoxicity of ADC aggregates. We constructed two anti-HER2 mAb-based ADCs that conjugated maleimide-C5-MMAE and had similar DAR values: wild-type human IgG1 (WT-MMAE) and human IgG1 with L234A/L235A mutation (LA-MMAE) (supplementary Fig. S3). The anti-HER2 mAb-based ADC aggregates induced by stirring stress showed different qLD profiles compared with T-DXd (Fig. 6a). We performed the reporter assays using FcγR-expressing reporter cells, and WT-MMAE aggregates showed FcγRIIa- and FcγRIIIa-activation properties which were lower than those of T-DXd aggregates, whereas LA-MMAE aggregates showed hardly any activation of FcγRs (Fig. 6b). Then, we evaluated the cytotoxicity of these ADC aggregates in FcγR-expressing reporter cells and MEG01-S cells. As shown in Fig. 6c, two anti-HER2 mAb-based ADCs (WT-MMAE and LA-MMAE) showed similar cytotoxicity in Jurkat cells regardless of aggregation as well as control (non-stressed) T-DXd and T-DXd aggregates. WT-MMAE aggregates showed higher cytotoxicity in FcγR-expressing reporter cells and MEG01-S cells than control WT-MMAE, though the enhancement of cytotoxicity of WT-MMAE aggregates was lower than that of T-DXd aggregates. Of note, LA-MMAE aggregates showed little enhancement of cytotoxicity in FcγR-expressing reporter cells and MEG01-S cells compared with control LA-MMAE, and the cytotoxicity of LA-MMAE aggregates was lower than that of WT-MMAE aggregates. These data indicated that the FcγR-activation properties of ADC aggregates affected the cytotoxicity of ADC aggregates in FcγR-expressing cells, and Fc-silencing could reduce the risk for off-target cytotoxicity of ADC aggregates in FcγR-expressing cells.

## Discussion

Off-target toxicity of ADCs is primarily dependent on the types of payloads (12, 13). For example, hematotoxicity, neurotoxicity, and liver toxicity have been reported as off-target toxicities of DM1-conjugated ADCs, including T-DM1. The mechanism of off-target toxicity of ADCs is considered to be the internalization of ADCs and/or the free payload released from ADCs into non-target cells. However, the mechanism of the internalization of ADCs into non-target cells is not clearly understood, and only a few studies have proposed potential mechanisms of internalization of ADCs in non-target cells. Though FcγR-dependent cellular uptake was one of the putative mechanisms of internalization of ADCs into non-target cells, the contribution of FcγRs on off-target toxicity of ADCs is still controversial. Upper et al. reported that T-DM1 internalization in differentiating megakaryocytes caused thrombocytopenia, which is a major off-target toxicity of T-DM1 (20). They also demonstrated the contribution of FcγRIIa to internalization of T-DM1 in megakaryocyte by using an FcγRIIa-blocking antibody and mutant trastuzumab with reduced FcγRIIa-binding affinity. In contrast, Zhao et al. reported that FcγRIIa did not play a critical role in the internalization of T-DM1 into megakaryocytes, while they agreed that the internalization of T-DM1 into megakaryocytes induced thrombocytopenia (21). In addition, though it is known that immunocomplexes and mAb aggregates are internalized via FcγRs and other receptors (22–24), the impact of aggregation of ADCs on internalization and cytotoxicity in non-target cells and the contribution of FcγRs to off-target cytotoxicity of ADCs and ADC aggregates has not been fully understood.

In this study, we assessed the impact of aggregation on cytotoxicity of ADCs in target cells and non-target cells by using two commercial trastuzumab-based ADCs. We generated ADC aggregates under harsh condition (stirring stress or thermal stress) to unravel the impact of aggregation on off-target toxicity of ADC aggregates (Fig. 1). Aggregation of ADCs reduced the killing activity of ADCs in HER2-positive target cells (Fig. 2a). In contrast, several ADC aggregates showed enhanced cytotoxicity in HER2-negative cells, especially FcγR-expressing immune cells (MEG01-S cells and THP-1 cells) (Fig. 2b). The enhancement of off-target toxicity was suppressed by filtrating the samples with 0.45 μm filter (Fig. 2). Though we considered fragment of ADCs or free payload induced by stress treatment had some contribution on off-target cytotoxicity, these results strongly suggested that aggregates, which were removed by 0.45 μm filter, were responsible for enhancement of off-target cytotoxicity. To examine the mechanism of off-target cytotoxicity, we focused on FcγR-activation properties of ADC aggregates. We demonstrated that the FcγR-activation properties of ADC aggregates were related to the internalization and enhanced cytotoxicity of ADC aggregates in FcγR-expressing reporter cells (Figs. 3 and 4). In addition, we revealed that FcγRIIa plays an important role in the cellular uptake of Tra-FL aggregates and cytotoxicity of ADC aggregates in a human megakaryoblastic leukemia cell line (MEG01-S) (Fig. 5), and Fc-engineering of ADCs for silencing of Fc-mediated effector functions reduced the off-target toxicity of ADC aggregates (Fig. 6). Our data indicated the involvement of FcγRs in the off-target toxicity of ADC aggregates, and the ADC aggregates which could be internalized via FcγRs had a major potential risk for off-target toxicity in FcγR-expressing cells.

Previous studies about the mechanism of thrombocytopenia of T-DM1 have reached conflicting conclusions in regard to the contribution of FcγRs on the off-target toxicity of T-DM1, as described above. In our study, we found that blocking of FcγRIIa did not affect the cellular uptake or cytotoxicity of non-stressed ADCs in MEG01-S cells (Fig. 5). In addition, the monomeric ADCs with different Fc regions (wildtype human IgG1 and the L234A/L235A variant) showed similar off-target cytotoxicity in FcγR-expressing cells, including MEG01-S cells (Fig. 6). Our results were in good agreement with the previous reports of Zhao et al., which suggested that off-target toxicity of monomeric ADCs was independent from FcγRs in differentiating megakaryocytes (21). On the other hand, we demonstrated that FcγRs play an important role for off-target toxicity of ADC aggregates by using FcγR-expressing reporter cells and an FcγR-blocking antibody. Additionally, the FcγR-activation properties of ADC aggregates with different Fc regions (WT-MMAE and LA-MMAE aggregates) affected the off-target cytotoxicity of these aggregates in FcγR-expressing cells. LA-MMAE aggregates that showed hardly any FcγR-activation properties showed little enhancement of cytotoxicity in FcγR-expressing cells. These data indicated that the FcγR-dependent off-target cytotoxicity of ADC aggregates reflected the FcγR-activation properties of these aggregates, and Fc-engineering for the purposes of silencing Fc-mediated effector functions (e.g., L234A/L235A substitutions) might reduce the potential risk for cytotoxicity of ADC aggregates in FcγR-expressing non-target cells.

It is known that some ADCs showed hepatic toxicity as adverse effects (12, 13), and Kraynov et al. reported that ADCs showed rapid and increased localization into hepatic cells, especially the Kupffer cells, compared with the naked mAbs (25). The mechanism of hepatic uptake of ADCs has been unclear, but it has been proposed that non-specific cellular uptake (especially macropinocytosis) contributes to the target-independent internalization of ADCs. Zhao et al. reported that macropinocytosis played an important role for ocular toxicity, thrombocytopenia, and neutropenia of ADCs, including T-DM1 (21, 26, 27). Based on the results of these studies, they considered that the charge or hydrophobicity-mediated unspecific interactions of mAbs—including ADCs—with cell surfaces were the trigger of an increase in pinocytosis. In their study, they revealed that the charge modification or polyethylene glycol (PEG) conjugation of ADCs modulated the cytotoxicity of ADCs in human corneal epithelial cells and human umbilical vein endothelial cells. These data are consistent with the phenomenon that hydrophobicity and charge affect the pharmacokinetics of ADCs (3, 28). We also agreed with their conclusion that macropinocytosis contributes to the cellular uptake of ADCs. However, the impact of aggregation of ADCs on cellular uptake by macropinocytosis is still unknown. Though it was reported that protein aggregates stimulated macropinocytosis in some cases (29), further studies will be needed in order to more fully understand the macropinocytosis of ADC aggregates. In our experiment, ADC aggregates showed biphasic dose–response cytotoxicity in TMNK-1 (an immortalized human liver endothelial cell line) regardless of the aggregation method and FcγR-activation properties (Fig. 2), indicating that there may be multiple cellular uptake mechanisms of ADC aggregates. Because HER2 and FcγRs are not expressed in TMNK-1 (data not shown), we considered that other receptors (such as C-Type lectin receptors and Toll-like receptors)- mediated endocytosis might contribute to the cellular uptake of ADC aggregates in TMNK-1 in addition to macropinocytosis. We also found that thermal stress-induced T-DXd aggregates, which did not activate FcγRIIIa, induced cytotoxicity in FcγRIIIa-expressing reporter cells (Fig. 3). There is a possibility that the aggregates possessed a weak FcγRIIIa-activation property, which could not be detected by our reporter assay but which was sufficient to induce off-target cytotoxicity in FcγRIIIa-expressing cells. In addition, the data also suggested the involvement of macropinocytosis or endocytic receptors other than FcγRIIIa. The mechanisms of cellular uptake and off-target cytotoxicity of ADCs and their aggregates seem to be more complicated than initially expected, and further studies will be required to elucidate them.

Though the unintended internalization of ADCs or free payloads have been considered as the mechanism of off-target toxicity of ADCs, as described above, another mechanism of off-target toxicity of T-DM1 was reported in a recent study. Endo et al. demonstrated that DM1 (the payload of T-DM1) interacted with CKAP5 on the cell surface in HER2-negative and HER2-positive cells, and the interaction of T-DM1 and CKAP5 induced Ca^2+^ flux and cell death (30). In the present study, we found that the non-stressed T-DM1 increased the luciferase activities in the FcγR-expressing reporter cells, but non-stressed trastuzumab and T-DXd did not (Fig. 3a). Because the NFAT-luc reporter used in our reporter cell lines is driven by Ca^2+^ signaling, we hypothesized that T-DM1 binding to CKAP5 and the resulting Ca^2+^ influx contributed to the reporter activation in FcγR-expressing reporter cells, though the expression levels of CKAP5 in Jurkat cells and FcγR-expressing reporter cells were not revealed. The reporter activation induced by non-stressed T-DM1 was inhibited by blocking of FcγR using Fab fragments of anti-FcγR antibodies (data not shown), and we now consider that FcγR may contribute to T-DM1-induced Ca^2+^ flux collaboratively with CKAP5 or other receptors. The impact of FcγR-activation induced by non-stressed T-DM1 on internalization and off-target toxicity of T-DM1 has not been elucidated, and further studies are needed to understand the off-target toxicity of non-stressed T-DM1.

Aggregation of biopharmaceuticals including ADCs has been well studied in the context of the development and quality control of biopharmaceuticals. In commercial biopharmaceuticals including ADCs, the amounts of HMW species (e.g., dimers) and sub-visible and visible particles having a particle size ≥10 μm are controlled by SEC and the light obscuration (LO) method, respectively. It was reported that sub-visible particles of protein aggregates with a diameter from 0.1 to 10 μm, which were difficult to evaluate by SEC and LO methods, also has a potential risk of immunotoxicity in the recent studies (10, 31). It was also reported sub-visible particles of protein aggregates were found in formulation condition or solution pass through after inline filter (16, 32, 33). It is known that the formation of mAb aggregates is affected by stress-inducing aggregation, the biopharmaceutical formulation, and mAb structure (11, 34, 35). The impact of ADC-specific characteristics, such as the structures of payload and linker, DAR, and conjugation site, on the aggregation of ADCs have also been well studied recently (36–39). On the other hand, studies evaluating the characteristics (e.g., FcγR-activation property) of ADC aggregates are still limited. In the present study, we evaluated the off-target toxicity and FcγR-activation property of ADC aggregates containing sub-visible particles, and demonstrated that the characteristics of ADC aggregates affect the potential for off-target toxicity. We found that the aggregates of three anti-HER2 IgG1-based ADCs (T-DM1, T-DXd, and WT-MMAE) showed different size distributions and different FcγR-activation properties, which could have impacted the off-target cytotoxicity in FcγR-expressing cells. These results suggested that not only the formation and amounts of aggregates but also their characteristics are important for the risk assessment of ADC aggregates. In regard to the control of the off-target toxicity of ADC aggregates, we demonstrated that Fc-engineering to silence the Fc-mediated effector functions was useful for reducing the FcγR-dependent off-target toxicity of ADC aggregates. However, as shown in our study, FcγR-mediated cellular uptake is a part of the mechanism of off-target toxicity of ADC aggregates, and further studies will be needed to clarify the relation between the characteristics and off-target toxicity of ADC aggregates. ADC aggregates used in this study were prepared by harsh condition, and the samples contained higher number of sub-visible particles. Their characteristics might be different from ADC aggregates in formulation condition. Therefore, further studies for evaluating the FcγR-dependent off-target toxicity of ADC aggregates which reflect the amounts and characteristics of ADC aggregates in formulation condition are important. In conclusion, our results demonstrated that aggregation of ADCs should be controlled much more carefully compared with the aggregation of other biopharmaceuticals, including mAbs, from the points of view of off-target toxicity and immunogenicity.

## Supplementary Information

ESM 1 (DOCX 126  kb)
