# Supplementary material for: Fcγ Receptor-Dependent Internalization and Off-Target Cytotoxicity of Antibody-Drug Conjugate Aggregates
Source: Pharm Res. 2021 Dec 27;39(1):89–103. doi: 10.1007/s11095-021-03158-x (PMC8837541; doi:10.1007/s11095-021-03158-x)
Supplement: Supplementary file 1 — (DOCX 126 kb) [file 11095_2021_3158_MOESM1_ESM.docx]

**Supplementary information**

**Fcγ receptor-dependent internalization and off-target cytotoxicity of antibody-drug conjugate aggregates**

Michihiko Aoyama^1,*^, Minoru Tada^1^, Hidetomo Yokoo^2^, Yosuke Demizu^2^, Akiko Ishii-Watabe^1^

*^1^Division of Biological Chemistry and Biologicals, National Institute of Health Sciences, 3-25-26 Tonomachi, Kawasaki-ku, Kawasaki, Kanagawa, 210-9501, Japan*

*^2^Division of Organic Chemistry, National Institute of Health Sciences, 3-25-26 Tonomachi, Kawasaki-ku, Kawasaki, Kanagawa, 210-9501, Japan*

**^*^Corresponding author**

Michihiko Aoyama, PhD

Division of Biological Chemistry and Biologicals, National Institute of Health Sciences, 3-25-26 Tonomachi Kawasaki-ku, Kawasaki, 210-9501, Kanagawa, Japan

Tel: +81-44-270-6515

Fax: +81-44-270-6517

E-mail: [aoyama-m@nihs.go.jp](mailto:XXX@nihs.go.jp)

***SI Table***

**Table S1. IC_50_ (nM) of trastuzumab, T-DM1, and T-DXd in HER2-positive or -negative cells**

|  |  | HER2^+^ cell | HER2^-^ cell | | | |
| --- | --- | --- | --- | --- | --- | --- |
|  |  | SK-BR-3 | Jurkat | MEG01-S | THP-1 | TMNK-1 |
| trastuzumab | native | 0.57 |  |  |  |  |
|  | stir | 5.19 |  |  |  |  |
|  | 90ºC | 13.90 |  |  |  |  |
| T-DM1 | native | 0.09 | 57.7 | 98.8 |  | 11.2 |
|  | stir | 0.29 | 37.0 | 15.7 | 5.6 |  |
|  | 90ºC | 2.96 | 73.6 | 225.1 | 12.4 |  |
| T-DXd | native | 0.18 |  | 194.8 | 498.6 | 126.8 |
|  | stir | 0.48 | 602.0 | 0.6 | 0.6 |  |
|  | 90ºC | 3.83 | 543.2 | 311.2 | 2.7 |  |

IC_50_ (nM) of trastuzumab, T-DM1, T-DXd was calculated with non-linear fitting (4-parameter logistic fitting). Diagonal lines mean IC_50_ could not be calculated with non-linear fitting.

***SI Figure***

***
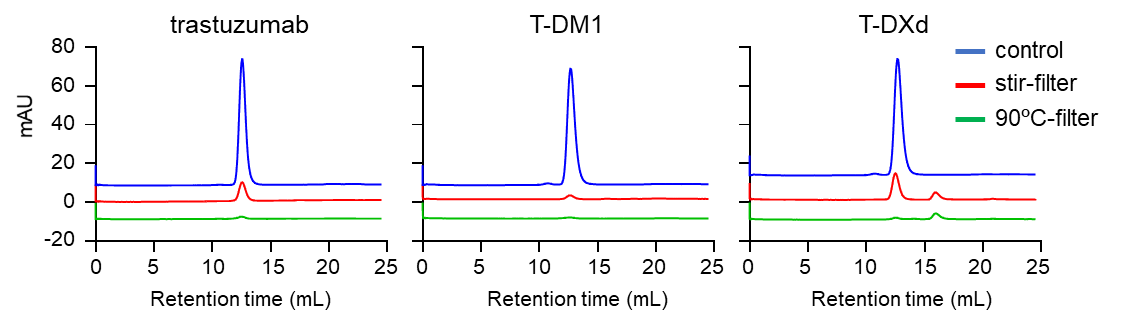
***

Relative amount (%) of each peak (vs total peak area of control)

|  | control | | | stir-filter | | | 90ºC-filter | | |
| --- | --- | --- | --- | --- | --- | --- | --- | --- | --- |
|  | HMWS | mono  -mer | fragment | HMWS | mono  -mer | fragment | HMWS | mono  -mer | fragment |
| trastuzumab | — | 99.6 | — | — | 15.5 | — | — | 2.2 | — |
| T-DM1 | 1.2 | 98.8 | — | — | 3.2 | — | — | 1.3 | — |
| T-DXd | 1.0 | 99.0 | — | — | 20.6 | 5.9 | — | 1.4 | 5.6 |

**Figure S1.** Size exclusion chromatography (SEC) analysis of filtered samples of ADC aggregates

The control (non-stressed) and filtered samples of stirring- and -thermal-stress induced aggregates of trastuzumab, T-DM1, and T-DXd were analyzed by SEC using a Superdex 200 Increase 10/300 GL column. The relative amounts of HMWS, monomer, and fragments in each sample were calculated from peak area. — indicated that relative amount was lower than 1.0% of total peak area of control samples.


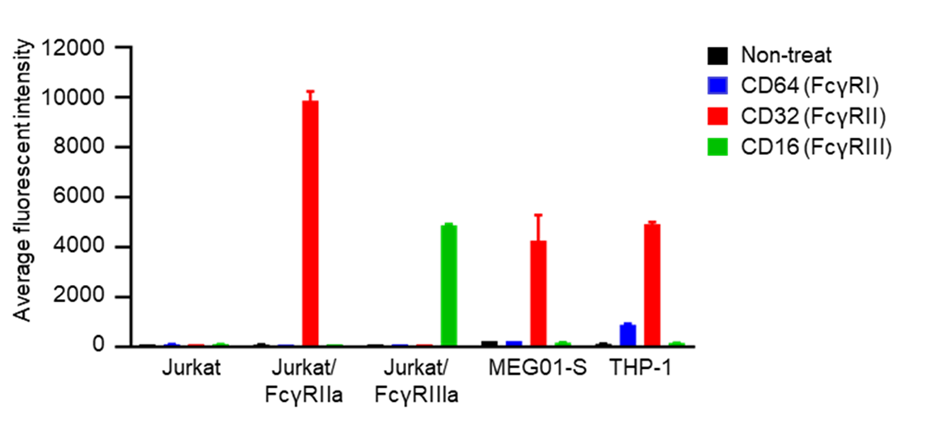


**Figure S2.** FcγRs-expression in the cell lines derived from immune cells

The expression of FcγRs (FcγRI, FcγRII, and FcγRIII) in Jurkat, Jurkat/ FcγRs/NFAT-Luc reporter cells, MEG01-S, and THP-1 cells was evaluated by flowcytometry analysis. The cells were stained with FITC-labelled anti-CD64 antibody, anti-CD32 antibody, or anti-CD16 antibody, and fluorescent intensities were measured by a flow cytometer. The data represent the means ± standard deviation (n=3).


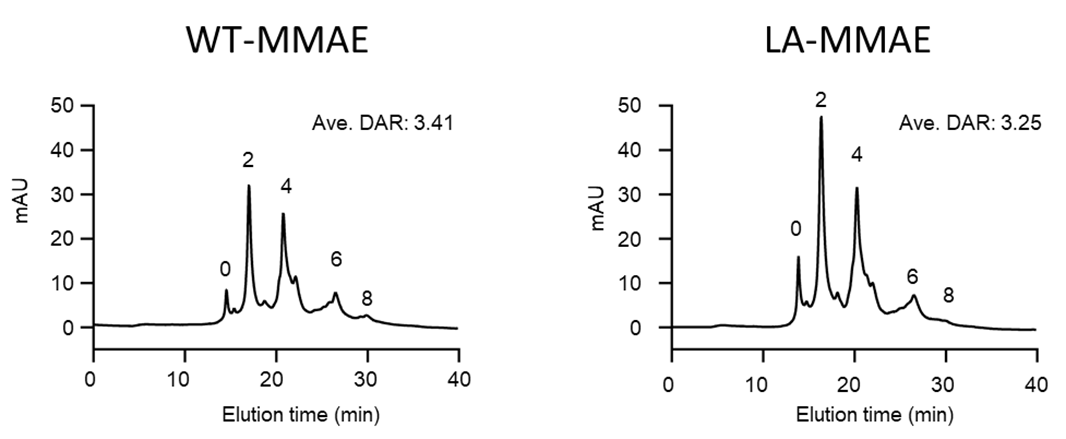


**Figure S3.** Hydrophobic interaction chromatography analysis of anti-HER2 mAb-based ADCs

The drug antibody ratios (DARs) of anti-HER2 mAb-based ADCs were analyzed by hydrophobic interaction chromatography (HIC) using a BSK-gel Butyl NPR column. The 5 peaks (DAR: 0, 2, 4, 6, and 8) were identified in HIC chromatograms of anti-HER2 mAb-based ADCs (WT-MMAE and LA-MMAE) obtained by a UV detector (280 nm). The average DAR was calculated from the area percentage in a chromatogram.
